# Pelvic Palpatory Tests in Manual Therapy and Osteopathy: A Critical Review of the Literature and Suggestions for New Research

**DOI:** 10.7759/cureus.64066

**Published:** 2024-07-08

**Authors:** Saverio Colonna, Marco Mazzanti

**Affiliations:** 1 Orthopedic Surgery, Spine Center, Bologna, ITA; 2 Education, Osteopathic Spine Center Education, Bologna, ITA

**Keywords:** refinement of palpatory perception, palpatory evaluation protocol, palpatory agreement, palpatory training, reproducibility of palpatory tests, reliability of palpatory tests, palpatory tests, somatic dysfunction, osteopathy, manual therapy

## Abstract

Manual therapists apply physical interventions to the entire structure of the body to promote healing, prevent pathologies, and improve patient health. In osteopathic practice, palpatory evaluation is considered a fundamental clinical practice requirement for identifying somatic dysfunction. Most of the articles published in this area have failed to demonstrate a level of reproducibility that supports palpation in evidence-based clinical practice. When considering the poor reliability of the palpatory tests highlighted in the literature, a discrepancy is noted with what is known about the tactile sensitivity of human hands. For static touch, the minimum size that can be detected, in the absence of applied movement or vibration, is approximately 0.2 mm. Yet, it seems that this high level of precision is insufficient to ensure reliability in the tests used to evaluate osteopathic somatic dysfunction.

The purpose that underscores this article is to determine how these two contradictory elements, high sensitivity and low reliability, can be present in palpatory tests. The article reports the literature findings regarding palpatory tests of pelvic, which is an important structure for clinical purposes. Additionally, a critical review of how these studies were conducted is provided to identify any elements that may justify the obtained results. Following recent accredited guidelines present in the literature, we propose suggestions on vision training methods, manual perception refinement training, the search for anatomical markers, and the position of the examiner in relation to the examinee that may be useful for future studies on the topic covered by the article.

## Introduction and background

Manual therapists apply physical interventions to the entire body to promote healing, prevent pathologies, and improve the patient's health [1]. In osteopathic practice, palpatory evaluation is a fundamental clinical practice requirement for identifying somatic dysfunction. Fryer, endorsing classical authors, claims that somatic dysfunction is detected by palpation if at least two of the four cardinal clinical criteria are present: tenderness, asymmetry, abnormal range of motion, and tissue texture changes [2-5]. The acronyms TART (Tissue Texture Change, Asymmetry, Restriction of Motion, and Tenderness) or ARTT are commonly used as a mnemonic aid to frame these four clinical signs. Over 40 years have passed since manual therapy researchers published the first work, to our knowledge, regarding the reliability of palpatory diagnostic tests, which are commonly used in manual therapists’ clinical practice (e.g., physiotherapists, chiropractors, and osteopaths) [6]. Most of the more than 200 related articles have failed to demonstrate a level of reproducibility that supports the use of palpation in evidence-based clinical practice [7]. Among scholars who applied rigorous scientific methods, most report that only some palpatory tests achieved reasonable reliability while most remain unreliable [8-11]. Several scientists since the early 1990s have found that, due to an insufficient number of well-performed studies, they cannot reach conclusions about the reliability of both static palpatory tests (for detecting the position of anatomical landmarks) and passive movement tests [12,13]. Consequently, the scientific community continues to question the relevance of manually performed diagnostic tests.

An emerging debate concerns reconceptualizing the value of manual examination and palpatory findings that are generally used by manual therapists, chiropractors, and osteopaths [14-16]. Palpation is a quick and inexpensive diagnostic modality, but it is also subjective. Empirical and theoretical gaps in the literature must be better articulated to establish the rationale for future studies and create a springboard for a renewed model based on palpatory findings and their use in manual therapies. Recommendations to promote the study of palpatory findings and conceptual models for manual therapies include employing qualitative research findings to generate a theoretical construct because qualitative results enable an improved understanding of practitioners' hypotheses while offering input on coherence and concordance, plausibility, generalizability, relevance, and applicability [17]. Renewing a model for the use of palpatory findings could enable practitioners to consider the growing understanding of a person-centered approach that is integrated with multidimensional patient profiles. The selective and informed use of these palpatory findings remains an important element in the clinical practice of manual therapy [14]. The objective of this article is to present a critical evaluation of the state of the art of palpatory assessment in manual therapy and to offer suggestions to improve reliability, credibility, and consequently, use in clinical practice.

## Review

Literature review

Haneline et al. reviewed 29 studies regarding the inter-examiner reliability of three palpation methods: finding the location of painful or tender points, landmarks, and position or alignment of the bony structures of the spine and sacroiliac joints [18]. They concluded that these three palpation methods generally exhibit low reliability. Therefore, although many pain palpation studies have reported acceptable K levels to Cohen's statistical test, no static palpation method has been proven to be clearly reliable. Seffinger et al. concluded that pain provocation tests are the most reliable but paraspinal soft tissue palpation tests are not reliable [19]. Stovall and Kumar, reviewing studies regarding the reliability of positional asymmetry assessments of bony anatomical landmarks in the lumbar spine and pelvis, concluded that for asymmetry assessments, the average inter-examiner positional reproducibility is slightly higher than random chance for all landmarks except for the medial malleolus, which exhibits fair reproducibility [20]. Both Stovall and Kumar and Seffinger et al. agreed that intra-examiner repeatability is greater than inter-examiner reproducibility [19,20]. Furthermore, Seffinger et al. report that neither the examiners’ discipline, their experience level, their agreement on the procedure used, nor the use of symptomatic participants increased reliability in the evaluation of standard palpatory landmarks [19]. Other researchers have come to the same conclusion: years of training and work experience do not improve reliability in palpatory findings [21,22].

Many within and outside healthcare professions have called for a broader evidence base for osteopathic medicine. Therefore, research must focus on the investigation of diagnostic tests, the evaluation and pathophysiology of somatic dysfunction, and the indications as well as physiological effects of osteopathic treatment. Beyond the manifest lack of inter-examiner reproducibility, validity has rarely been studied [23]. This is a critical situation because diagnostic validity, which is based on external criteria that refer to a gold standard, indicates how well the test actually evaluates what it intends to evaluate. Validity and reliability are often used interchangeably, but they represent different elements.

Validity is the accuracy with which a measurement records the true state of a phenomenon, while reliability measures the agreement, consistency, or repeatability of results [24,25]. However, a measurement may be consistent and reliable but not necessarily valid. The typical example that represents this phenomenon is that of several arrows that continually hit a small area of ​​the target, but fail to hit the center of the target, which represents the objective. Kmita and Lucas note that “the field of diagnostic accuracy has been labeled in the British Medical Journal as the 'new frontier'” [21]. Despite the lack of evidentiary support, a typical manual therapy examination involves the detection of altered symmetry (e.g., pelvic torsion) and a determination of the clinically relevant side through positional asymmetry, palpation of motion, or other testing procedures that may inform the force vector of the correction [26]. Asymmetrical positions of the posterosuperior iliac spine (PSIS) in a pelvic assessment, for example, may imply opposing rotations of the innominate bones, where the bone on the side of the inferior PSIS has rotated posteriorly relative to the other side, which in turn has rotated or is believed to have rotated anteriorly [27].

Neurophysiological indices of tactile perception

When considering the poor reliability of the palpatory tests highlighted in the literature, some discrepancy is noted regarding what is known about the tactile sensitivity of human hands. In neurophysiology and psychophysics, the perceptual threshold (a measure of sensitivity) is the perception level below which a sensory stimulus is not noticed. For each of the five senses, absolute perception thresholds are defined on an empirical basis [28]. Sight: perception of candlelight 50 km away on a clear and calm night; hearing: perception of a mechanical clock 6 m away in a silent room; taste: 1 t of sugar in 3 L of water; smell: a drop of perfume spread throughout the entire volume of three rooms; and touch: the pressure of a bee's wing dropped from a height of 1 cm.

In absolute terms, could human hands have the ability to perceive the pressure of a bee's wing and yet be unable to objectively discriminate greater tension in the paraspinal muscles or in the positional asymmetry of the vertebral processes or iliac spine? For static touch, the minimum amplitude that can be detected, in the absence of applied movement or vibration, is approximately 0.2 mm [29,30]. Can humans discriminate, solely through touch, between two structures that differ by a single layer of surface molecules? Researchers have asked this question and have conducted studies to investigate humans’ ability to discriminate between surfaces based only on the surface’s chemistry [31]. The findings have indicated that properly trained individuals can quickly refine the forces and sliding speed needed to distinguish between surfaces that differ by a single molecular layer [31].

Critical analysis of the literature

Comparing the minimum threshold of tactile perception with the studies regarding the reliability of palpatory tests, one perceives a discrepancy; consequently, we consider published findings to identify where these contradictions may arise. The hypotheses problems that we found and explored enable us to answer the aforementioned doubts and can be summarized in three points: 1) assume that the hands have an innate level of perceptive ability that is useful for manual diagnosis; 2) do not consider all elements involved in the palpatory diagnosis; and 3) the researchers did not correctly follow the current guidelines in developing this type of study.

Adequacy of training for manual diagnosis

For the studies in this group, we questioned whether the quantity and quality of the typical number of training hours are adequate for those who use their hands as evaluation tools [7]. Many assume that the evaluator’s hands, which inherently have a high perceptive sensitivity, are already trained for required palpation tasks. Cooperstein and Hickey conducted a systematic review of 13 studies published from 1985 to 2008 regarding the reliability of PSIS sampling as a palpatory landmark of the pelvis; data were reported using Cohen's Kappa statistical calculation [32]. The findings prove that none of the studies reached the level K≥0.40, which defines a moderate agreement, while a substantial agreement (K≥0.60) is believed to identify a clinically useful procedure [33]. Of the studies included in the review: seven studies assessed the perceptual ability of expert therapists; three studies assessed expert therapists and students; and three students assessed only. None of the studies reports the training path that the examined subjects followed. Palpation is a complex and fundamental skill for those who heal with their hands, but it is a difficult task to teach and learn. Learning complex motor skills can be simplified by reducing cognitive load. Simplifying the task by dividing it into smaller parts allows beginners to handle each part separately [34,35]. Since an incorrect technique is difficult to change later, it is preferable to ensure the quality of motor skill learning in the early training stages [36].

In the review cited above, three studies evaluated the reproducibility of palpatory tests in chiropractic and osteopathy students but did not reach a K level of agreement; hence, the conclusion reached by the article’s authors is that students do not reliably perform palpation [37-39]. However, it would be more correct to conclude that the students evaluated perhaps did not receive adequate training to achieve palpatory reliability; in other words, one should question the students’ training more than palpation overall. The same could be said for the expert examiners who participated in the studies included in the review [32]. Before becoming experts, they were students whose type of training probably did not differ from that received by the students still in training. Consequently, one may wonder that if expert palpation is not reliable for evaluating osteopathic dysfunctions, the diagnosis from which the therapeutic strategy derives, then how can we justify the clinical results? In their daily work, experts use thinking strategies that are largely influenced by their ability to perceive large and meaningful patterns. Conversely, beginners can only recognize smaller and less developed patterns [40].

Feltovich et al. argue that competence constitutes an adaptation, and its development is intimately associated with the ability to gather a broad set of skills, knowledge, and mechanisms that monitor and control cognitive processes to perform efficiently and effectively within a specific domain [41]. Experts are then able to restructure, reorganize, and refine their representation of knowledge, skills, and actions to operate effectively in the workplace. Returning to the previous question, therefore, the hypothetical answers can be multiple: 1) experts do not rely only on palpation to diagnose but integrate many elements; 2) patients’ level of dysfunction is not comparable to that of the healthy subjects evaluated included in the studies cited; 3) to make the experimental evaluation scientifically correct, strategies are often used that are not comparable to clinical practice. For example, to prevent recognition of the tested subject in experimental studies, which is useful for intra-examiner comparison, part of the subject is covered, and this involves subtracting many elements that are useful to the expert eye when diagnosing. In a subsequent paragraph, training models are presented to increase the hands’ perceptive ability, which is useful for palpation.

The terminology of "palpatory tests"

The term typically used to indicate this type of evaluation is palpatory tests. Speaking generally about palpatory tests, many hold the erroneous belief that this type of evaluation is based on the ability of stereognosis. Stereognosis, also known as tactile perception or tactile gnosis, is the ability to perceive and recognize an object’s shape without visual or auditory information; rather, only tactile information is used to provide cues about texture, size, spatial properties, and temperature [42]. As previously reported, some authors argue that somatic dysfunction is detected by palpation if at least two of the four TART cardinal clinical criteria are present [2]. The definition of palpatory tests focuses on one’s ability to appreciate physical conditions using hands alone. In clinical practice, this is inaccurate. Take, for example, the evaluation of asymmetry and alteration in the range of movement at the PSIS level, which is a diagnostic test used by various professionals (manual physiotherapists, osteopaths, chiropractors, posturologists, etc.) in their clinical practice. The hands seek contact with the anatomical point to be evaluated, but any symmetry or asymmetry is truly detected by the eyes. Mitchell underscores the importance of vision in some osteopathic tests without mentioning the fundamental role of postural evaluation; however, it seems that these indications have not been considered by researchers or teachers [43,44]. A systematic review evidences a positive effect of the eyes on tactile acuity for parts of the body in which vision has a plausible functional connection [45]. In the palpatory test of the pelvic landmarks, the actual diagnostic aspect is entrusted to vision rather than hands. In fact, the hands only enhance the spatial position of the anatomical landmark, but the eyes provide the spatial information to the brain to ultimately elaborate the diagnostic response. Furthermore, sight also has a role in evaluating the degree of tissue tension or stiffness. For example, when an indenter, such as the examiner’s fingertips, contacts a compliant object, the examiner’s view provides information regarding the time course and deformation pattern of the object's surface and the area around the contact region. While tactile information is informative for softness perception, visual information provides indirect cues from which softness can be inferred but not perceived. A study from approximately 10 years ago was performed to investigate, using a complicated technological apparatus, whether the softness of objects with deformable surfaces can be deduced from indirect visual information only, and if so, how this indirect visual information is integrated with direct tactile information [46]. The results highlight that the softness of natural deformable stimuli can be inferred through vision alone. This secondary visual information is integrated with the primary tactile information in visuotactile judgments that contribute to the final judgment by approximately 35%.

Taking these data into consideration, therefore, it would be more accurate to define this type of manual evaluation as a visual-palpatory test. However, if the eyes participate in or even dominate the diagnosis, then how much time is dedicated to visual training during preparation for palpation? Identifying whether an imaginary line that joins the hands or fingers positioned in the two anatomical reference points, for example, the PSIS, is horizontal or non-horizontal is difficult and cannot be assumed. Consequently, it is important to dedicate time to specific visual training before conducting research studies on the reproducibility of visual-palpatory tests and, even better, before training manual therapists. Furthermore, some ocular disorders should be excluded from participants, such as metamorphopsia, a disorder first described by Foster and defined as a distorted vision in which a grid of straight lines appears wavy and parts of the grid may appear empty [47]. This visual disturbance sometimes precedes the clinical appearance of maculopathy [48]. An assessment of visual abilities to exclude spatial plane identification disorders is indicated for anyone practicing manual therapy.

Methodological scrutiny of studies

The methods with which the studies present in the literature were performed are subjected to critical scrutiny. Patjin, whose work is highly recommended for anyone approaching this research field, provides guidelines for conducting studies on the validation of manual tests [49]. From these guidelines, we highlight important points relevant to our critical review: number of examiners, choice of examiners, choice of subjects examined, agreement phase, time dedicated to training to refine perception and agreement, and training methods.

Number of Examiners

The first point Patjin highlights is the erroneous tendency to believe that the more observers agree on a diagnostic procedure, the better the reproducibility properties of that procedure [49]. This assumption is based on a serious logical error: reproducibility studies are primarily intended to provide information on all aspects of a procedure’s repeatability and reproducibility. This means that, essentially, the number of observers involved has no relationship with the level of reproducibility in a study that addresses diagnostic procedures. Therefore, only two observers are needed in a diagnostic reproducibility study if only the reproducibility property is being assessed.

Before starting a reproducibility study, the examiners involved must adequately agree, through specific training, on all the diagnostic procedure's execution details and its final judgment. If, however, the study is intended to evaluate the effect of the training and will implement different training phases in the study protocol, then it is advisable to use more than two observers as participants. Of the 13 works reviewed by Cooperstein and Hickey, three studies used only two examiners [32,38,50,51]. Therefore, the remaining studies do not truly evaluate the test's repeatability but rather the effectiveness of the agreement training that was used and how such training was actually performed.

Choice of Examiners

Many reproducibility studies involve observers with different expertise levels. These levels are a predictive or explanatory factor for the level of kappa coefficients (K) that the examiners report as belonging to the study's categories. The same objections as previously stated regarding the idea of using more than two observers in a study apply to using examiners with different skill levels in reproducibility studies. Patjin emphasizes that reproducibility studies are primarily intended to provide information on all aspects of a diagnostic procedure's reproducibility properties; this means that the examiner's level of competence has essentially no relationship with the procedure's reproducibility [49]. There is a tendency to believe that experts have greater results than those who are less experienced or still in training because experts have more palpatory tactile sensitivity and discriminative ability. This belief has been supported by some authors, while others do not confirm it [8,21,22,52-54].

If multiple observers with different experience levels participate in a reproducibility study, especially if they have not passed a training phase of the protocol, then the final kappa coefficient obtained reflects, for example, more of the experienced observer's personal interpretation and the less-experienced student's understanding of the procedure's evaluated diagnostics instead of the procedure's reproducibility. Expert therapists, in fact, over the years of their profession, unconsciously develop their personal interpretation of the execution and judgment of a diagnostic procedure. Consequently, their procedure may differ from the standardized procedure described in the literature. Moir et al. note that all therapists develop their own criteria by which to determine the standards of a testing procedure and that there may be differences in the interpretation of results as well as difficulties in objectively measuring these results [8]. Therefore, one should expect that the results of studies that are intended to demonstrate experience-related palpatory accuracy have mixed results [13,19,37]. For students, however, the lack of experience with the palpatory diagnostic procedure can influence the final result, which is quantifiable with Cohen's K coefficient. When designing a study regarding palpatory repeatability, one must consider that the examiners should remain the same in the initial evaluation, agreement training, and final post-training evaluation, as proposed by Patjin [49]. This measure has not been implemented by all studies in the literature [7,55,56].

Choice of Subjects Examined

Haas recommends that reliability studies use a representative sample of subjects observed in clinical practice because symptomatic subjects should provide a uniform distribution of negative and positive results; additionally, the kappa statistic becomes unstable when there is a limited variety of results [25]. Haas' statement is certainly valid when analyzing palpatory tests to diagnose medical pathologies [57]. Some authors in the osteopathy field, however, have suggested that somatic dysfunction, the main objective of osteopathic palpatory tests, may exist in asymptomatic individuals, simply creating altered mobility and predisposing them to an imbalance and consequent pathology with symptoms in the same location or in other regions [58-60]. Considering these claims and anecdotal classroom experience that pelvic asymmetry is common in asymptomatic students, the inclusion of asymptomatic subject groups is justified in the studies considered in Cooperstein and Hickey's review [32]. From a practical standpoint, a rounded number of 40 was selected to make these types of reproducibility studies relatively simple and cost-effective [49]. Generally, statisticians advise that basic reproducibility studies that have dichotomous outcomes and use kappa statistics should have approximately 40 subjects. Modern sample size calculations based on statistical power are recommended to determine appropriate study sample sizes.

In our recent study on the reproducibility of the palpatory evaluation of the stiffness of the iliotibial band (ITB), we followed the indications of the literature and evaluated 40 subjects as a sample size, without a specific power calculation [40,56]. The post hoc analysis, considering an alpha of 0.05, the power calculation, dividing the results of the stiffness difference between the two sides into seven levels, was a beta of 93.82%; when divided into three levels, the power increased slightly to the beta of 94.55%. In statistical power analysis, the acceptable power (1 beta) is typically set at 0.80 (80%), which means that there is an 80% probability of correctly rejecting a false null hypothesis. This corresponds to a beta level of 0.20 (20%), which represents the probability of a type II error (failing to reject a false null hypothesis). This threshold is generally considered an appropriate balance between sensitivity and practicality in research studies. As can be seen, the level of beta achieved in the aforementioned work was well beyond what was considered acceptable [56]. Therefore, we can confirm that the number of 40 subjects to be included in this type of study is an adequate number for the required statistical power.

Agreement Phase

Before starting a reproducibility study, observers must, regardless of their personal expertise, agree on all details of the performance of the diagnostic procedure and its final judgment. In the event that only the reproducibility properties of a palpatory test are evaluated, this agreement can be acquired by introducing a training phase into the study protocol.

Training Phase for Each Observer Regardless of Skill Level

Only after completing the agreement or training period is the performance and judgment standardization of a diagnostic procedure guaranteed. Osteopathy students, as well as those in other musculoskeletal system disciplines, such as chiropractic, manual therapy, and massage, receive in-depth theoretical and practical training in palpating muscles, bones, joints, and connective tissues to diagnose altered functional states that can be manually treated. Patjin considers the agreement search phase to be essential to reproducibility studies because this phase enables the foundations for a positive result [49]. The first elements of the training period are dedicated to observers' mutual agreement on all detailed aspects of the performance and on the final judgments of the diagnostic procedure they want to evaluate. The ideal way to train the diagnostic procedure is to have both observers participate in a session in which they perform the diagnostic procedure on the same topic. For palpatory evaluation, observers must seek agreement on the following aspects: the precise point at which the anatomical marker to be evaluated is taken, the intensity of the push, and the level at which the sampling of the two sides should be considered symmetrical and asymmetrical. The comparison between the mutual performances of the diagnostic process and the definition of each examiner's final judgment must lead to an unambiguous execution of the diagnostic process. One of the first studies to our knowledge in which examiners tested the importance of preparatory training for a correct evaluation regarding the agreement of a palpatory test is by Gerwin et al., in which the authors studied the amount of improvement that could be achieved in inter-examiner repeatability of the manual assessment of myofascial trigger points by introducing a three-hour agreement training session between the examiners before sampling [61]. The level of inter-examiner agreement increased from an assessment of poor by both examiners in the pre-training to acceptable in the post-training. Although O'Haire and Gibbons conducted a one-hour training session immediately preceding the sampling, it is unclear whether the degree of asymmetry for which the examiners defined an asymmetrical result was discussed [37]. One of the fundamental points for further investigation when searching for an agreement is when to consider the points taken to be symmetrical or asymmetrical. Agreement between examiners can be increased by standardizing what constitutes the minimum difference in symmetry rather than leaving such judgments to the examiners' discretion. Asymmetry can be assessed in millimeters if a positional aspect of the anatomical reference points is evaluated, which is more complex if other palpatory aspects such as stiffness are evaluated. Inter-examiner agreement may also be increased if examiners receive more training than just immediately before starting the study.

Time Dedicated to Training to Refine Perceptions and Agreement

The literature offers disparate indications regarding the time dedicated to finding agreement: O'Haire and Gibbons propose one hour; Fryer et al., however, increase the time to three hours; and Consorti et al. recommend nine hours [37,39,62]. Degenhardt et al. use a greater number of hours: one or two hours per week for a period of four months, which, when added together, ranges from 16 to 32 hours [7]. The number of hours proposed by Degenhardt et al. is significantly higher than other authors who used training to refine manual perception and agreement between examiners; hence, it is necessary to underscore that the types of evaluation were greater than those used by O'Haire and Gibbons and Fryer et al. [37,39]. In fact, the tests concerned the four general types of palpatory diagnostic assessments commonly taught and used in clinical practice (TART), including osteopathy. Degenhardt et al. used eight palpatory samples per subject: 1) differentiation of the tissue characteristics (texture), 2) evaluation of the positional asymmetry of the reference points in static conditions in the three planes of space, 3) evaluation of the positional asymmetry of the reference points in dynamic conditions in the three planes of space, and 4) evaluation of tenderness [7]. The final results indicate a significant improvement in interobserver reliability, which is within the clinically acceptable range (K=0.40) for the tenderness tests (baseline sample K=0.32; final sample K=0.68, p=0.02) and tissue structure (baseline sample K=0.12; final sample K=0.45, p=0.003) [7]. Although inter-examiner reliability improved significantly for positional asymmetry in the transverse plane (baseline sample K=0.17; final sample K=0.34; p=0.001), the threshold for adequate reliability was not reached (K=0.40). Adapting the test for movement asymmetry in the transverse plane to the anteroposterior translational elasticity of the lumbar spinous processes improved interobserver reliability (baseline sample K=0.10; final sample K=0.20, p=0.004); however, even on this parameter, a clinically significant level was not reached. Degenhardt et al. conclude that training for consensus in palpatory tests is effective in significantly improving inter-examiner reliability but does not provide achievement of a clinically acceptable level [7]. It must be stressed that each of these points requires manual skills and specific training as well as the use of different methodologies and tools. Therefore, the number of hours used by Degenhardt et al., which is higher than in other works, may have been insufficient to reach an acceptable level simply due to the greater amount of manual skills required [7]. Indeed, Denslow clarifies that each of the TART elements can be palpated and evaluated separately; additionally, students should begin the evaluation process by thinking of these as separate activities and then later merging them into a single procedure [63]. Degenhardt et al. further report a possible confounding factor in the quantity of assessments performed on each subject by the three examiners [7]. In phase 3, each vertebra was tested 18 times by examiners. Moreover, the examiners noticed that the movement characteristics changed regularly between examiners' evaluations. The authors believe that this experience supports the hypothesis that tests in which a movement is present that stimulates the sensory nervous system can cause neuromotor reflexes to adapt to the stimuli, especially in young individuals (as with the study's subjects), causing changes in results after repeated stimuli. Can we exclude that this phenomenon does not occur when the evaluation is static and free of movement?

Returning to the hours of training, the question that arises is the hours of necessary training to examine the reproducibility of a palpatory test. The answer is provided by Patjin who, in his detailed guide article, reports that the hours cannot be programmed a priori but must be continuously updated with intermediate verification samples [49]. After the baseline sampling, intermediate samplings are performed with a smaller number of subjects compared to the baseline; only after establishing a priori the minimum level that must be reached for the Cohen's K index can the final sampling be conducted. If a failure occurs in the intermediate samplings, then the training hours must be increased. Patjin recommends that the number of subjects in the intermediate sampling should be calculated incrementally. In other words, the control evaluation uses 10 subjects without particular inclusion and exclusion criteria; if the agreement reached is acceptable to the authors and K≥0.6, then the next sample should use 20 [49]. If an acceptable level is not reached in the intermediate evaluation, then the training hours should be increased.

It should be noted that, according to Patjin, manual tests must have a dichotomous response to confirm or exclude a possible pathology; consequently, he believes that it is useful to include the prevalence index (P-index) of the pathology being investigated in the statistical evaluation [49]. In palpatory evaluations, especially osteopathic ones, the presence or absence of pathology is not diagnosed; rather, the presence or absence of dysfunction, which often manifests itself with asymmetries, is sought [39]. Dysfunctions are the basis of osteopathic evaluations; consequently, it is preferable to use the samples from these tests not in dichotomous mode, since the human body is not a physiologically symmetrical structure, but in ordinal mode, providing a gradation in multiple levels [17,64,65].

Training Methods

The preparatory training for the evaluation is divided into two modalities: 1) sharpening perceptive skills and 2) achieving inter-examiner agreement on the sampling method and results interpretation.

Sharpening perceptive skills: The use of correct teaching methodology is essential to maximize students' palpatory skills [66]. In most osteopathy schools, the curriculum for the first three years focuses on educational material presented through lectures and laboratory sessions. Training in palpatory anatomy is typically conducted in laboratory environments where students work on each other under the teacher's guidance. Students acquire cognitive knowledge from didactic presentations of anatomy, biomechanics, and dysfunction, combining this information with subjective evaluations of experts (teachers or assistants) applying the technique. Students perform the technique by attempting to imitate the movements demonstrated and receive verbal feedback from the expert, ideally immediately after the technique is performed so that students can easily compare the feedback with their memory of executing the technique [15,67]. This feedback is critical for students to learn which components of a technique were not adequately applied [68,69]. Students thereafter generally repeat the technique in unsupervised settings. Theoretically, this process creates skilled and confident practitioners who have developed preprogrammed action patterns, or engrams, to reduce the need for high mental effort when performing palpatory skills, thereby allowing interventions to be applied accurately and efficiently within the clinical context [70,71]. A comparison of the complexity of skill performance with the subjective observational aspects of the current model for teaching manual skills reveals that the educational process can be layered with substantial ambiguity or variations in perception between students and instructors [72]. The widely used modalities make it difficult for students to receive the objective feedback they need regarding whether they are feeling what they should be feeling. Teachers represent the gold standard of reference, but as per some study results in the literature, expert therapists, whether physiotherapists or osteopaths, may not actually demonstrate greater palpatory perceptive ability [18]. It is frustrating for students not to perceive or to perceive the opposite of what they report the teacher to have perceived. The underlying physiological processes involved in manual diagnosis and osteopathic manipulative treatment (OMT) are complex and require high levels of sensory and motor processing as well as coordination [73]. Considering the human brain's plastic nature, one can argue that developing palpatory diagnostic skills is likely associated with adaptive behavioral, neuroanatomical, and neurophysiological changes. Acquiring skills in a specific domain of professional practice is, however, a long and arduous process. There is consensus among skill development researchers that a human needs approximately 10000 hours of intense practice to become an expert within a chosen domain [74]. Teaching and assessing manual diagnostic skills with methodologies that allow students to coherently interpret the complex sensory input and motor control necessary to perform techniques from a scientific perspective is not completely realistic in osteopathy schools and in all manual therapy training courses. However, using the scientific reductionist model, modern instrumentation could revolutionize the teaching and performance of palpatory skills by enabling instructors to provide objective, timely, and repetitive feedback within the training process [75]. Researchers have demonstrated considerable value in using instrumentation to provide objective feedback to students during manual therapy training [15,67,76,77]. For example, objective feedback for a high-velocity low-amplitude technique for the lumbar, cervical, and thoracic areas demonstrates that several biomechanical parameters can be consistently changed during 25% of routine chiropractic training compared to the standard course [67]. One explanation for why quantitative measurement feedback appears to improve palpatory skills comes from motor learning. It has been reported that trainees must characterize both kinematic and kinetic components to coordinate the performance of complex skills [78]. The instrumentation could provide this type of feedback to students immediately during the initial training session but also on a recurring basis as students practice the techniques. In the current teaching model, feedback, which is crucial to successful instruction, is often compromised due to limited time spent on palpation. However, quantitative data that define optimal parameters for performing osteopathic techniques are not yet available, so efforts should be made to codify them. Only through serial and unbiased observations can we learn more about the important aspects of palpation that have established the value of osteopathic diagnostic and therapeutic palpation since its inception. This process will not be quick or easy.

**Figure 1:**
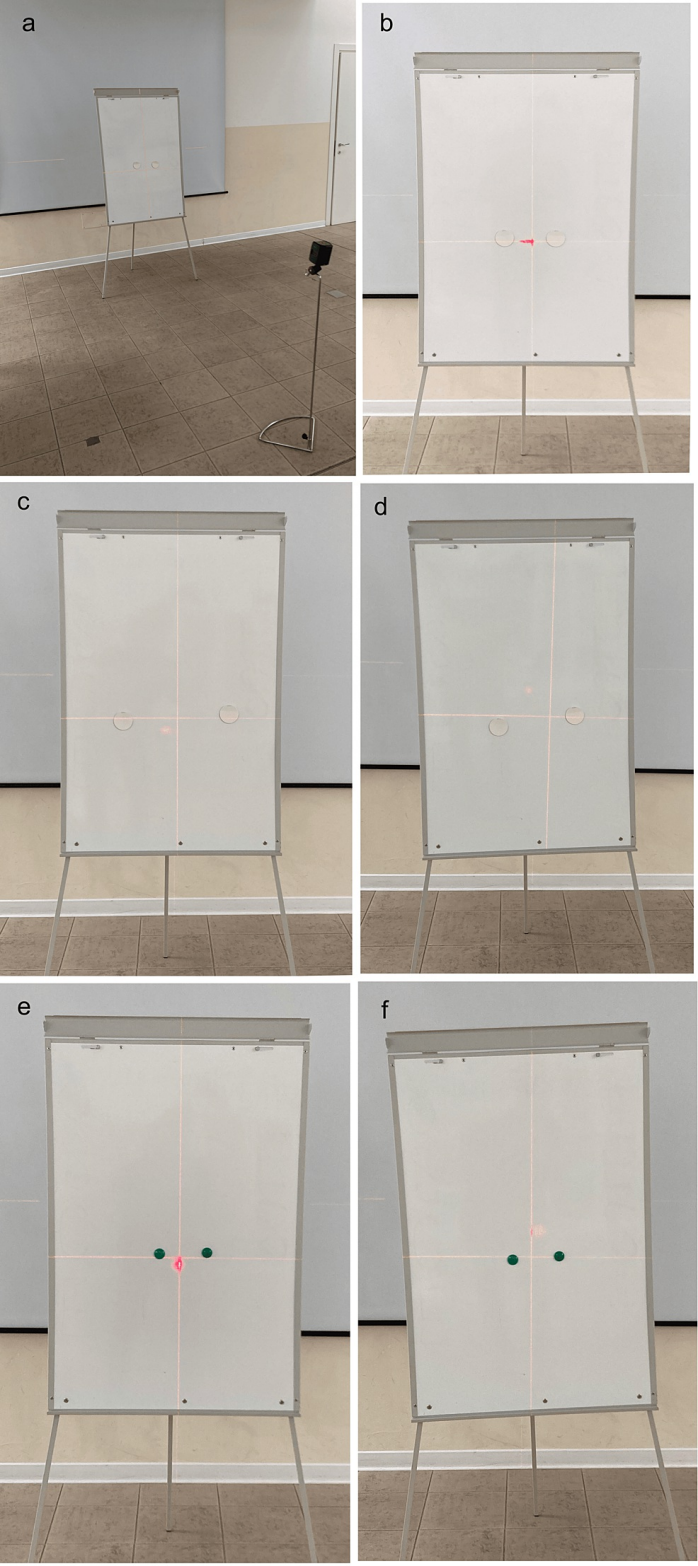
Magnetic whiteboard and magnets A magnetic whiteboard and magnets are used to train vision in the spatial recognition horizontality and verticality: a) position of the whiteboard and self-leveling laser device, b) use with the horizontal whiteboard and large magnets at a distance of approximately 10 cm, c) horizontal whiteboard and magnets spaced 30 cm to 40 cm apart, d) oblique whiteboard and large magnets, e) horizontal whiteboard and small magnets spaced approximately 10 cm apart, and f) small magnets and oblique board. Image credit: Colonna, S

Various training protocols and methods to improve palpatory skills have been described in the literature [7,39,79-81]. One of the first methods, which lasts approximately one hour and is divided into seven phases, is called PALPATE (P- Palpate, A-Anatomy, L-Level, P-Purpose, A-Ascertain, T-Tweaking, and E-Evaluate or normalize) and is based on cognitive and motor learning theories that were developed and proposed by Aubin et al. [80]. Researchers have also evaluated the response in identifying pelvic asymmetries (in the PSIS and greater trochanter) in healthy volunteer subjects [55]. They artificially elevated the lower limb with a 2 cm wedge hidden under the heel and observed 76 examiners, comprising osteopathy students and teachers, before and after two types of training (PALPATE and tailored training), each of which lasted one hour. The results indicate better results in improving examiner performance, especially with the PSIS markers, among the group who underwent the tailored training compared to both the group who did not undergo any particular training and the group who underwent the PALPATE training. The peculiarity of tailored training is the examiners' involvement in completing the preparation program, which involved one hour of further refinement training, during which the suggestions received by the examiners after the first sampling experience were included. This increased the examiners' interest level, which was an important factor in improving the second sampling detections. At the Osteopathic Spine Center Education, we hypothesized that an objective, immediate, standardized in vitro method for palpation assessment and training would lead to an improvement in skills compared to the classic training provided by the osteopathy training curriculum in Italy. To adequately train examiners to perform reliable palpatory tests, we considered the importance of vision in palpatory tests, determining that the training hours should be divided into those dedicated to vision and those dedicated to pure palpation. In the palpation laboratory, we designed and introduced dedicated visual training exercises that have been employed for some years. One of these involves using a magnetized whiteboard on which we position magnets and a self-leveling laser beam (Figure 1a) spaced approximately 3 m from the magnetic board.

It is possible to increase the difficulty in relation to how the board is positioned as well as the size and the positioning of magnets. We start with the whiteboard positioned with a perfectly vertical major axis and the two magnets positioned in the whiteboard's center on a horizontal (Figure 1b) or vertical axis. The student must identify the presence of horizontality or verticality or quantify the difference in height. The magnets are positioned on a horizontal axis at a distance of approximately 10 cm. Using the laser beam without the student seeing, the magnets are positioned on the horizontal axis defined by the laser light or on an oblique axis. The laser is turned off and the subject, positioned at a distance of 2 m, must look at the magnets and identify whether they are at the same level or whether they differ by a size larger than the diameter of the magnet. After the student answers, the laser is turned on again and the correct answer is displayed. Normally it starts with the difference of a magnet, and the student must determine whether the difference in height between the two magnets is of a size equal to or less than or greater than the diameter of the magnet; this process is repeated several times, turning the laser on and off and moving the magnets, after three correct answers, the student can then identify whether the difference in height between the two magnets is equal to or less than the diameter of half a magnet; after three correct answers, the student can move on to 1/4 of a magnet. The difficulty can be increased by distancing the magnets (Figure 1c), making the whiteboard oblique (Figure 1d) so as to alter the horizontal and vertical reference points, or using smaller magnets while maintaining the same progression with the whiteboard (Figures 1e, 1f). We also recommend performing this progression model with the magnets arranged vertically.

Various methods and materials can be found in the literature to evaluate and improve palpatory sensitivity and skills, ranging from simple handcrafted instruments to more expensive and technologically sophisticated variants [56,79,82-89]. To make the result of palpation as objective as possible, technological models and simulations are gaining an increasingly important role in medical training. The virtual haptic back (VHB) development was undertaken to address these limitations. The VHB is a virtual reality simulation of the human back's mechanical properties that is an aid for teaching clinical palpatory diagnosis [89]. It simulates the human back's contours and tissue structure and is presented to users both tactilely (by touch) and graphically (by sight). The simulation is based on measurements of real dorsums, contours captured by 3D photography, and tissue structure measured as tissue compliance with a PHANToM 3.0 haptic interface (SensAble Technologies, Woburn, MA) that was used as a force-displacement probe. A pilot study with 89 volunteer subjects who were osteopathic medical students demonstrates that VHB practice improved their ability to detect regions of altered compliance on the VHB [87]. During a preliminary test they were, on average, only able to identify regions that differed in compliance by at least 40%; after eight VHB practice sessions, they detected regions that differed in compliance by only 11%. Anonymous ratings provided by students or users indicate that these practice sessions were useful in their clinical laboratories as they learned to palpate regions of altered tissue structure on their fellow students. The simulation provides immediate feedback on whether users' diagnoses are correct, which students reported was not provided optimally in traditionally structured labs. Based on these results and the recommendations of osteopathic manipulative medicine teaching staff, VHB has been incorporated into the curriculum of some osteopathic universities [87].

Agreement between the examiners on the sampling method and on the interpretation of the results: Regarding the evaluation of positional palpatory tests such as those of the pelvis, much depends on how the tests are performed. Patjin recommends that observers begin the initial session by performing the diagnostic procedure on one another [49]. The following diagnostic components should be thoroughly discussed to ensure standardization of the entire diagnostic process: placement of the left and right hands or fingers, the position of the observer, the position of the subject/patient, the direction of the passive or active motion, anatomical landmarks for the directed motion, and description of the final judgment.

Typically, humans manually explore the stiffness of an object by repeatedly inducing imprints on the object's surface over time [90]. We know that fingers differ in the ability to produce force and in the perception of stiffness, which seems to be connected to the size of the fingertip and to differences in the spatial density of mechanoreceptors [91,92]. Furthermore, fingertip sensory sensitivity correlates with the cortical brain representation of the fingers in S1 and Brodmann 3b and 1, which suggests a neural basis for systematic differences between the fingers [93]. The literature reports that fingers differ in their sensory ability to perceive pressure, in spatial acuity, and in the two-point discrimination threshold [94]. In these studies, analyzing localization and two-point discrimination in glabrous skin revealed better performance from the thumb, index, and middle fingers compared to the ring and little fingers [94]. The little finger appears to have the worst localization threshold. Similar responses are also found in the active and passive perception of object volume [95]. Authors have wondered whether performance differences between fingers can explain spontaneous use patterns, so they studied natural exploration behavior in softness discrimination tasks, which is an important modality for evaluating fascial systems, to identify which fingers or finger combinations are used spontaneously [96]. The subjects analyzed preferred to use only one finger in 38% of the exploration phases, two fingers simultaneously in 33%, and three fingers simultaneously in 25%; rarely were four (3%) or five (1%) fingers used simultaneously. The index and middle fingers were most frequently used, which were followed by the ring and little finger, respectively. The thumb was not included in the evaluation.

**Figure 2:**
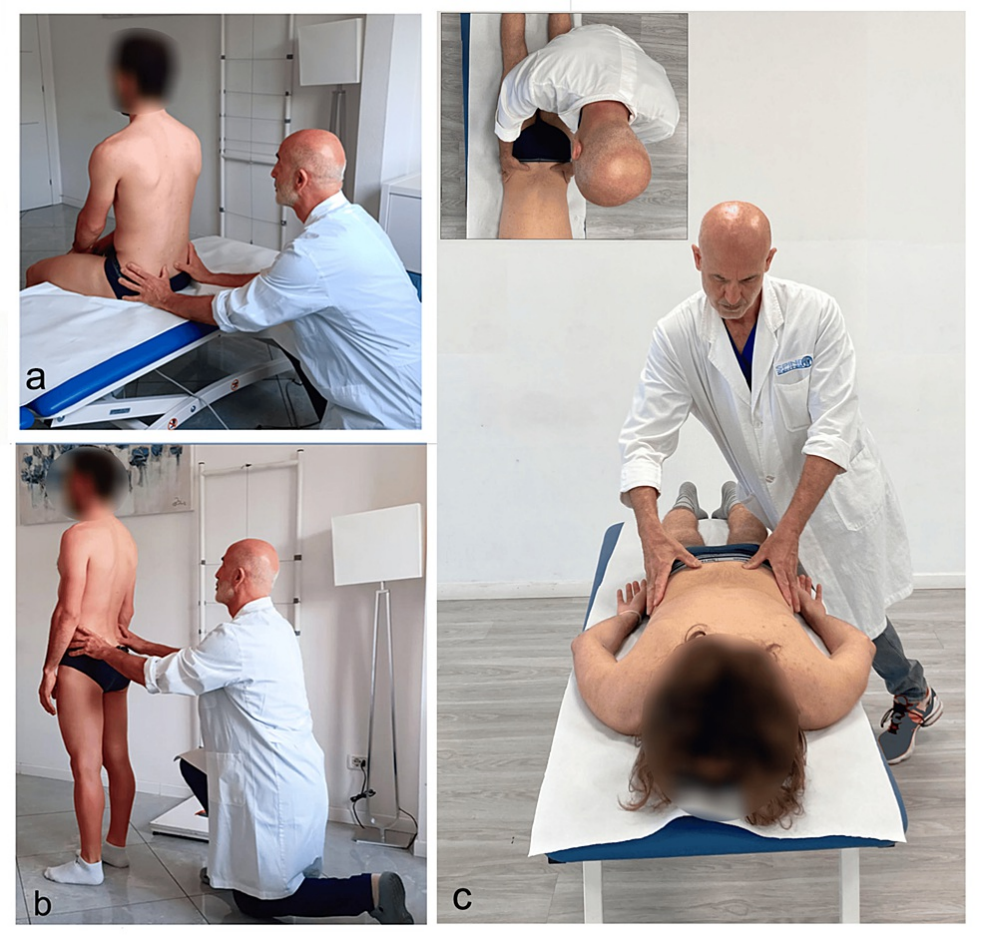
Classic method for height assessment of the PSIS Evaluation of the PSIS with the subject in seated (a), standing (b), and prone position (c). Note the difficulty in aligning the examiner's eyes, head, trunk, and pelvis with the examinee’s body in the prone position. Image credit: Colonna, S. PSIS, posterosuperior iliac spine

In practice, the sampling of anatomical points and, above all, the visual evaluation of the spatial level change is based on how they are performed: orthostatic position, sitting, prone, and supine. In the evaluation with the subject sitting (Figure 2a) or standing (Figure 2b), the examiner positions himself frontally or posteriorly with his shoulders parallel to those of the patient and aligns his eyes with the points to be evaluated (level of PSIS, anterosuperior iliac spine (ASIS), iliac crests, etc.). For pelvic assessments, the subject is in a prone or supine position (Figure 2c), and the examiner's position is typically next to the table.

**Figure 3:**
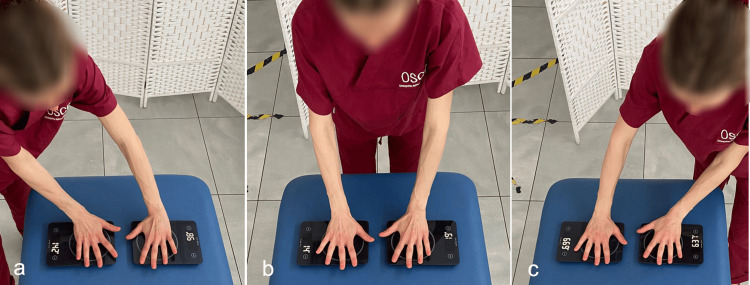
Evaluation of the symmetry of the hand pressure exerted using common scales Examiner positions in relation to the scales are a) on the right side, b) in front, and c) on the left side. Image credit: Colonna, S

As recommended by osteopathic authors and subsequently adopted in some research studies, the examiners stand on the side of the table that corresponds to their dominant eye-examiners with a right-dominant eye standing on the right side of the patient, unbalanced above the table in search of a vertical eye position on the landmarks to be evaluated [37,39,97,98]. This slanted position leads to a reduction in tactile and visual perception capabilities. Janda supports the existence of oculo-pelvic reflexes that cause any modification of pelvic orientation to affect the eye position [99]. Additionally, the eye position changes the perception of muscle tone, particularly in the suboccipital muscles. The implications of changing the eye position, due to the position of the pelvis or head, add multiple factors to consider when attempting to ensure optimal observed palpatory efficiency [100]. A recent study investigates the perceptual acuity of the hands when the object to be evaluated is in front (hands are in front of the eyes, head, and body) and when the object is at the side (hands are to the side of the eyes, head, and body) [101]. The results highlight a performance improvement when the hand is aligned with the direction of the eyes, head, and body and with the direction of the stimulus diagrams submitted to the participants, in other words, in a more congruous postural condition. These results suggest that tactile spatial perception sensitivity may utilize an eye-, head-, or body-centered reference rather than a hand-centered reference. Therefore, if the subject to be examined is lying prone, which is the position used by O'Haire and Gibbons, then the myofascial component is more relaxed and, therefore, allows better contact with the bone markers, such as the PSIS; however, it conditions the visual evaluation and palpation, thus making it more imprecise [37]. The increased precision of manual assessments made with the assessment objective positioned in front of the examiner's eyes, head, and body is demonstrated in the results of unpublished thesis studies, carried out in the palpation laboratories of the OSCE school of osteopathy. The objective of the study was to evaluate the thrust symmetry and the ability to perceive stiffness using three different hand positions in relation to the body with 49 students. In the first study, we aimed to identify the body’s spatial position that would allow the examiner’s hands to be pushed as symmetrically as possible on common scales. The assumption that underscored this study is this: to bilaterally evaluate the difference in stiffness of myofascial systems, such as the paravertebral muscles, the push in two hands or fingers must be symmetrical. This is confirmed in the literature, which reports a correlation between the thrust symmetry and the discriminative capacity of lumbar dysfunctions. In this study, the student stood in three different positions in relation to the scales (on the right side, in front, and on the left side) (Figure 3) and performed three pushing intensities (mild, medium, and intense) with the palm of his open hand resting on the scale, eyes closed (therefore, in blind mode regarding the result) [102].

**Figure 4:**
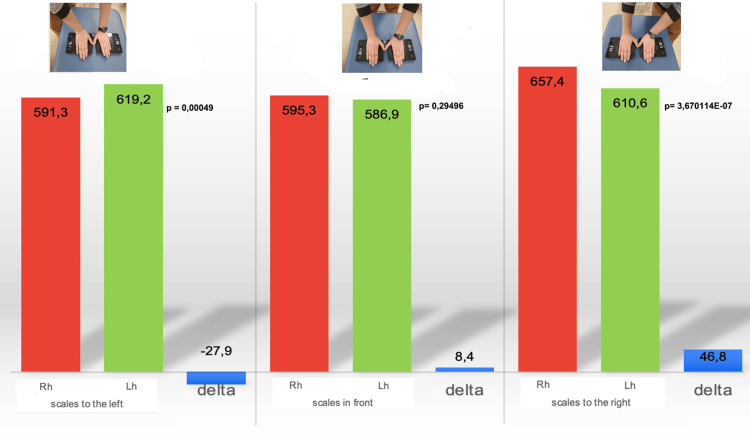
Difference in thrust between the two hands in three different positions Histograms represent the thrust expressed by the right (red column) and left (green column) hands,  adding the results of the three push intensities, in the examiner's position to the right, in front, and to the left of the scales. The blue columns represent the delta of the force value expressed by the left hand minus that of the right hand. The t-test value of the comparison between right and left hands is also reported. Image credit: Colonna, S.

As illustrated in Figure 4, the difference in thrust between the two hands, adding the results of the three push intensities, is smaller in the front position; additionally, the comparison with the student's t-test highlights a difference that does not reach statistical significance and does not appear with the other two positions.

**Figure 5:**
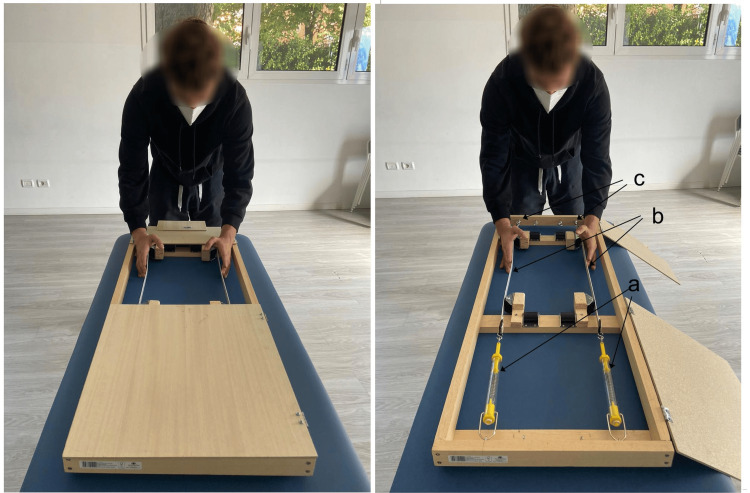
Pretendo We handcrafted the Pretendo instrument to determine the tension of the string (b) using tie rods (c); the tension is quantified by spring dynamometers (a). Image credit: Colonna, S.

The second objective was to evaluate whether the different body positions regarding the evaluation objective affected the evaluative perception. Using an instrument that we handcrafted (Pretendo) (Figure 5), we evaluated the discriminative capacity of the tension, which was managed by tie rods, of two strings connected to dynamometers to enable us to determine a known tension.

**Figure 6:**
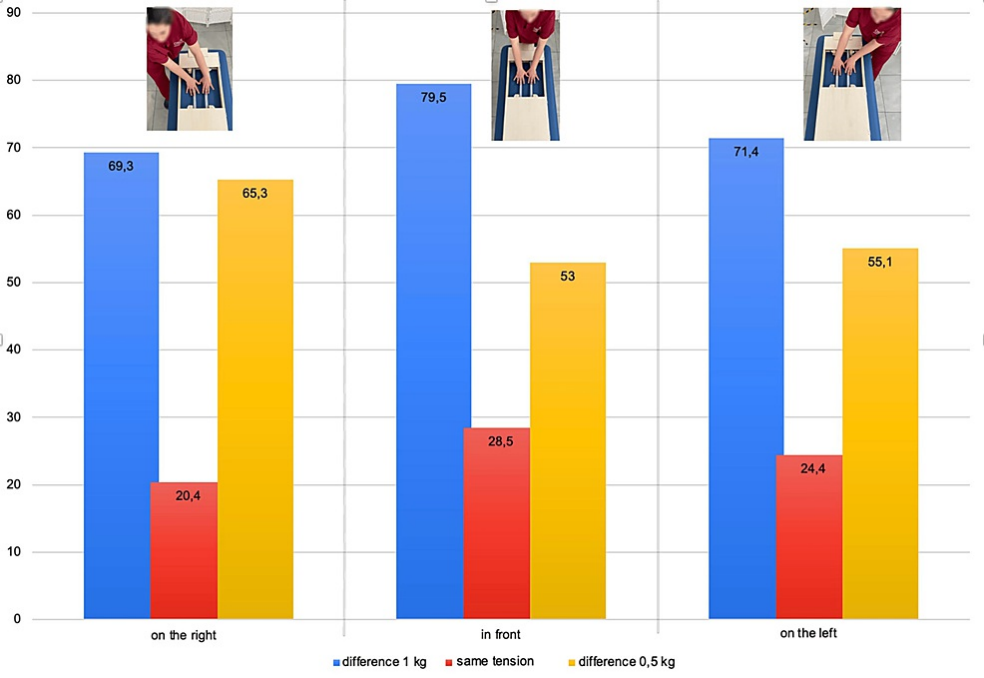
Pretendo testing results from three examiner positions Graphic representation of the percentage of correct answers regarding the string stiffness in the examiner's position to the right, front, and left of the Pretendo. The blue column represents a 1 kg difference between the strings; the red column indicates a 1 kg difference between the strings at the same tension, and the yellow column represents a 0.5 kg difference between the strings. Image credit: Colonna, S.

The evaluation occurred in the same three examiner positions as used in the previous research instrument: on the right, in front, and on the left. We randomly evaluated three tension levels that were preset by the tie rods: a difference of 1 kg, 4 kg, and 3 kg between the two strings; no difference between the strings (both set at 4 kg); and a difference of 0.5 kg, 4 kg, and 3.5 kg. The possible responses of the examiner performing the blind assessment were increased tension on the right, equal tension, and increased tension on the left. When the difference between the two strings was 1 kg, we observed the highest percentage of correct answers in all three positions; the central position reached the highest percentage (79.59%) compared to both the examiner on the right (69.39%) and on the left (71.43%) (Figure 6).

**Figure 7:**
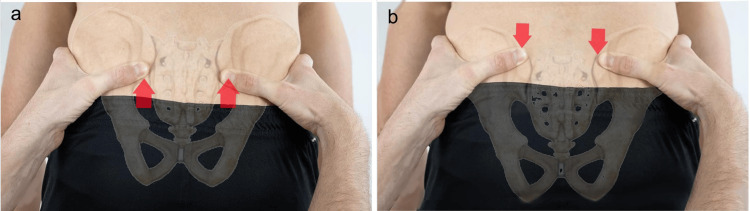
Representation of the PSIS marker assessment method Representation of the manual mode of PSIS sampling as classically proposed (a) and modified by Cooperstein [32,108] (b). Image credit: Colonna, S. PSIS, posterosuperior iliac spine

For the other two surveys, the percentage of responses dropped significantly. With the same string tension, the examiner positioned in front of the Pretendo reveals the highest percentage of correct answers (28.57%) compared to both the examiner on the right (20.41%) and on the left (24.49%). When the difference in tension between the two strings dropped to 0.5 kg, the rating with the examiner on the right reached a higher level (65.31%) than those with the examiner on the left (55.10%) or in the center (53.06%). We attempted to explain this unexpected result, but even considering the different hypotheses, it remains incomprehensible. Aggregating the data for the different tensions revealed no difference, although at 0.5 kg and 1 kg, the front evaluation received 54% of correct answers, whereas the examiner on the right received 52% of correct answers and the examiner on the left received 50% of correct answers. These data confirm the French et al. findings [101]. Fryer's work, in which 10 examiners evaluated the spatial position of the ASIS, PSIS, medial malleoli, and inferior lateral sacral angle in 10 subjects in the prone and supine positions, only the PSIS during seated flexion exhibited a higher Cohen's K value in the evaluation of the malleoli, although it did not reach sufficient agreement [39]. These data could certainly be due to a greater ease in finding the anatomical landmarks, as the malleoli are in fact easier to locate than the PSIS and ASIS, but here again, we cannot exclude that the examiner's position may have played a role in the different evaluation outcome. Additionally, the PSIS evaluation during the seated flexion test demonstrated only a slight agreement between examiners, although the test was conducted with the examiner and examinee aligned with shoulders parallel. In this case, we attribute the poor result to the greater difficulty in correctly finding the PSIS. As stated previously, the advantage of the lying position is that the myofascial systems are relaxed, which allows the bony landmarks to be reached and perceived more easily, but it places the examiner lateral to the subject being evaluated. During trunk flexion in standing and sitting positions, however, the tension of the long dorsal ligament (which is part of the sacrotuberous ligament in the PSIS) effectively inserts the hamstrings in the sacrum and the PSIS (muscles that manage the anteversion of the pelvis) and induces a departure from the bone plane, especially from the lower edge of the PSIS, which is the sampling area recommended by osteopathic and non-osteopathic authors [97,98,103-107]. To reduce this increased tension, which moves the fingertips away from the bone plane, the examiner often increases the thrust, causing the patient to lose balance. Therefore, Cooperstein et al. propose using as a landmark not the lower part of the PSIS (Figure 7a), as proposed in the literature, but the upper part (Figure 7b) [32,105,108].

**Figure 8:**
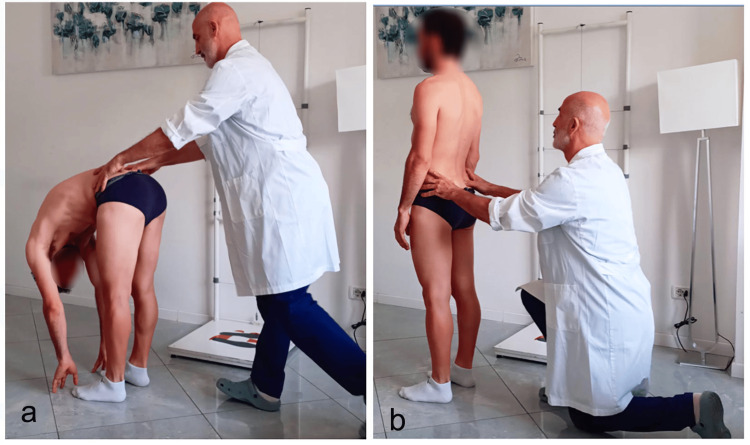
Modified standing flexion test The images illustrate the modified standing flexion test: a) initiation phase and b) arrival phase. Image credit: Colonna, S.

In this way, he also believes that the execution of the test proposed by the aforementioned author, defined as sitting-standing, is useful for demonstrating the existence of a discrepancy in the lower limb. In the literature, researchers have proposed evaluating PSIS movement starting in the neutral position (first detection) and following it until the end of flexion (second detection) for both the standing and sitting flexion tests [3,59,105,109]. The test is to be considered positive on the side in which the PSIS, comparing the first and second measurements, seems to move more cephalically and ventrally. From our experience, however, during the sitting and standing flexion tests, it is more reliable if the recording moments of the PSIS sampling are reversed. The symmetry of the PSIS in a flexed position (first detection) (Figure 8a) is detected, as the examiner maintains fingertip contact with the thumb under the PSIS, and followed as the patient rises, extending the trunk, to the neutral position (second detection) (Figure 8b).

As noted previously, the difficulty in conducting the test is in maintaining contact with the bony landmarks during flexion. The first sampling is performed when the patient is in the starting flexion position, a position which makes the detection of PSIS slightly more difficult, but it is conducted in a static condition, which is easier and facilitates the possibility of following the PSIS as the patient returns to a neutral position. In research on the palpatory repeatability of some tests, always with reference to the sampling method, biases can arise that alter the results. When evaluating intra-examiner repeatability, one of the fundamental requirements is that the examiner must not recognize the subject being evaluated because it limits or cancels the bias of remembering what occurred in the previous evaluation. Often, however, the methods used to hide the identity of the subject being evaluated alter the perceptive ability. For example, an unpublished study was performed at the OSCE osteopathy school palpation laboratory regarding an evaluation of the stiffness of masticatory muscles, such as the temporalis and masseter. The question arose regarding whether it was better to perform the evaluation with open eyes, which could create the bias of intra-examiner repeatability, whereas blindfolding the examiner could create a bias by altering the sample because, as previously explained, sight has an essential role in palpation; furthermore, the evaluation of these muscles is performed by the examiner who assists in the diagnosis with vision. We surveyed the examiners who participated in some of these studies on the reproducibility of palpatory tests, and all remarked on the near impossibility of remembering the data after evaluating only a few test subjects. Consequently, we recommend when performing these types of measurements, considering the difficulty of completely eliminating bias and the lesser importance of intra, as opposed to inter-examiner repeatability, that an evaluation that is not conditioned by recognition of the test subject should be performed with the use of vision.

**Figure 9:**
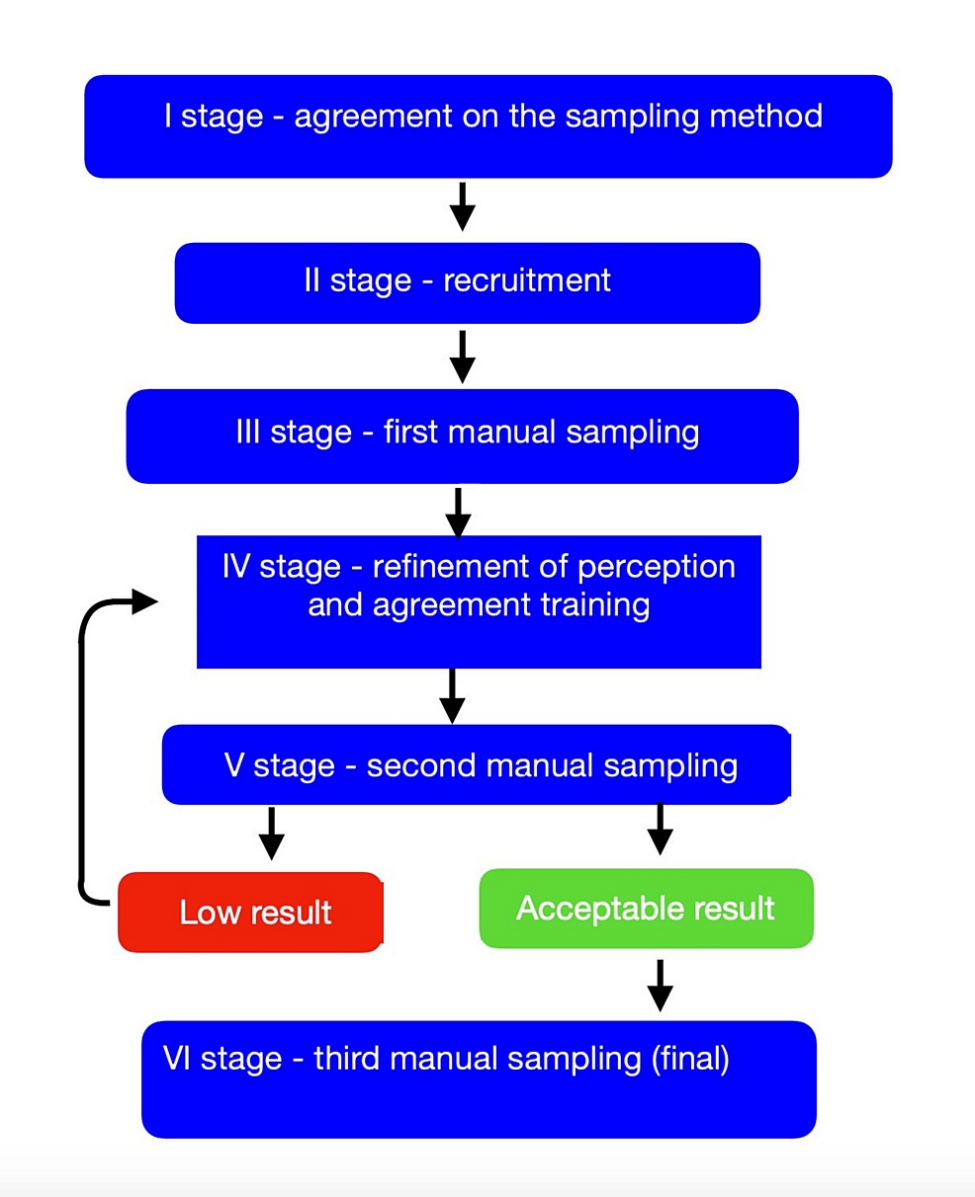
Flow chart of planning a reproducibility study The flowchart illustrates the necessary steps for a study that investigates the reproducibility of palpatory tests. Image credit: Colonna, S.

Agreement on how to interpret the results: Taking the pelvic evaluation as an example, once the PSIS height is identified, the examiners must agree on which delta of difference falls within a condition of symmetry as well as how to stage the difference, following the example of recent work [56]. Establishing the maximum range of difference to be considered symmetrical is an important element of training. Erroneously considering the human body to be a symmetrical system leads to thinking that the reference element of health is symmetry, but this is not true [110]. In the context of asymmetry, one must consider to what extent the body can compensate, as establishing not only whether an asymmetry is present but also quantifying this asymmetry could be useful for diagnostic and therapeutic purposes. To our knowledge, only one of our studies fully adheres to the guidelines proposed by Patjin: we assessed the reliability of directly palpating the ITB as an alternative to the Ober test [49,56]. Two examiners followed the protocol outlined in the diagram (Figure 9), evaluating 40 subjects for ITB tension, which was categorized into three levels (greater tension on the right, equal tension, and greater tension on the left) and seven levels (very tense, medium tension, and slightly more tense on the right; equal tension; and very tense, medium tension, and slightly more tense on the left).

The results achieved in the final sampling are as follows: for the intra-examiner agreement with three levels of tension, the average between the two examiners was K=0.965; for the inter-examiner agreement with three levels of tension, the average of the two measurements was K=0.872. The results for the evaluation of tension with seven levels are as follows: for the intra-examiner agreement, the average of the two examiners was K=0.911, while the inter-examiner agreement was K=0.759. These data are different from those reported in the literature. It should be noted that the ITB, being a more superficial structure with a small subcutaneous layer, is more easily accessible than, for example, the PSIS; nevertheless, the examiners underwent a 20-hour training to refine skills and agree on the single tension parameter of this anatomical structure.

## Conclusions

Manual palpation is an ancient practice that is widely employed by healthcare professionals in their daily practice. However, several works in the literature have highlighted a lack of reliability in palpatory examination results, both in validity and reproducibility. This article critically examined potential reasons for the poor reliability observed in the literature, despite the acknowledged significant sensory capacity of the hands. The findings underscore the need to review the palpatory skills teaching model applied in training schools. By accurately analyzing how palpatory tests are conducted, evaluators should recognize the crucial role of vision in these examinations. Indeed, vision's role is sufficiently significant that it may warrant a change in terminology from palpatory tests to visual-palpatory tests. Therefore, both tactile and visual abilities should be refined.
